# Loss of proteasome subunit RPN-12 causes an increased mean lifespan at a higher temperature in *C. elegans*

**DOI:** 10.17912/micropub.biology.000234

**Published:** 2020-04-06

**Authors:** Lourds Fernando, Victoria Nguyen, Tyler Hansen, Andy Golden, Anna Allen

**Affiliations:** 1 Department of Biology, Howard University, Washington DC; 2 Laboratory of Biochemistry and Genetics, National Institute of Diabetes and Digestive and Kidney Diseases, National Institutes of Health, Bethesda, MD; 3 Presently- Department of Biochemistry, School of Medicine, Vanderbilt University, Nashville, TN

**Figure 1:**
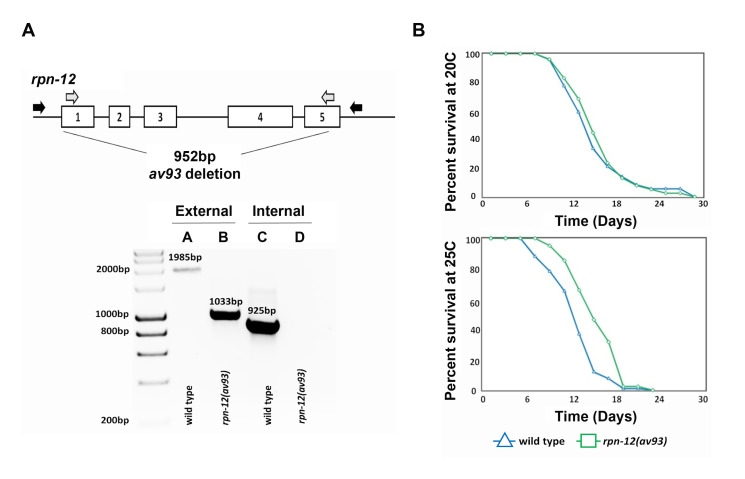
*rpn-12(av93)* mutant lifespan comparison to wild type at 20**°**C and 25**°**C. (A) Schematic of the *rpn-12* gene showing the 952bp deleted region made via CRISPR/Cas9 genome editing. Exons are numbered 1-5 (external primer regions shown in black arrows and internal primer regions shown in grey arrows). Agarose gel image showing PCR amplification of the *rpn-12* gene in wild type (Lanes A and C) and *rpn-12(av93)* (Lanes B and D) animals using external primers (Lanes A and B) or internal primers (Lanes C and D). (B) Lifespan analysis of the *rpn-12(av93)* mutants compared to wild type at 20**°**C and 25**°**C (single trials). Lifespan was determined by assaying percent survival of the hermaphrodites from the L4 stage in age synchronized larvae for wild type (n=71 at 20**°**C and n=92 at 25**°**C, shown in blue lines) and *rpn-12(av93)* mutants (n=76 at 20**°**C and n=41 at 25**°**C, shown in green lines). There was no significant difference between the lifespans of wild type and mutant animals at 20**°**C (*p* value 0.6163). There was a significant difference in the mean lifespans of wild type and mutant animals at 25**°**C (*p* value 0.0001). Log-Rank statistical test to determine mean lifespan and Mann Whitney U test to determine maximum lifespan was performed using OASIS2 software (Han *et al.*, 2016). Lost animals were not included in the statistical analysis.

## Description

The 26S proteasome is one of the major proteolytic machineries in cells and is composed of nearly 33 different subunits (Budenholzer *et al.*, 2017; Chen *et al.*, 2008; Papaevgeniou and Chondrogianni, 2014). The subunits are arranged as one or two 19S regulatory particle(s) capping a cylindrical catalytic 20S core particle (Budenholzer *et al.*, 2017). Each subunit is highly conserved from yeast to mammals, and proper function of the proteasome is crucial for survival of organisms (Papaevgeniou and Chondrogianni, 2014). Spatiotemporal expression of specific subunits and their roles in regulating the proteasome activity is still not clearly understood.

RPN-12 is a subunit of the 19S regulatory particle (RP) of the 26S proteasome in *Caenorhabditis elegans* (Boehringer *et al.*, 2012; Takahashi *et al.*, 2002). In *C. elegans,* loss or down regulation of a single proteasome subunit causes embryonic lethality*,* with the exception of three of the 19S RP subunits: RPN-10, RPN-12, and DSS-1 (Keith *et al.*, 2016; Pispa *et al.*, 2008; Shimada *et al.*, 2006; Takahashi *et al.*, 2002). While the roles of RPN-10 and DSS-1 were previously studied, more detailed investigation into the function of RPN-12 has not been performed*.* A heterozygous mutant containing a 952bp deletion of the coding region of *rpn-12* was generated [*rpn-12(av93)*] using CRISPR/Cas9 genome editing technology. The heterozygous *rpn-12* null mutant was then homozygosed and determined to be homozygous viable with apparent fertility issues. Only 36% of the *rpn-12(av93)* hermaphrodites produced progeny due to sperm production defects (this phenotype will be published elsewhere). Since efficient proteasome activity is important for the longevity of most organisms, we wanted to investigate whether *rpn-12(av93)* mutants may have an altered lifespan compared to wild type animals (Saez and Vilchez, 2014; Vilchez *et al.*, 2012). To determine whether the lifespan of *rpn-12(av93)* mutant animals was affected, lifespan assays were conducted at 20**°**C and 25**°**C. The mean lifespan of *rpn-12(av93)* hermaphrodites (15.94 days) was not significantly different from wild type hermaphrodites (15.54 days) at 20**°**C. Interestingly, at 25**°**C the mean lifespan of *rpn-12(av93)* hermaphrodites (15.59 days) was increased compared to wild type animals (12.80 days). However, the maximum lifespan of both *rpn-12(av93)* and wild type hermaphrodites was not significantly different at 25**°**C. The lifespan analysis at 20**°**C was performed three times (cumulative wild type n=240 and mutant n=192) and at 25**°**C was performed twice (cumulative wild type n=185 and mutant n=135). Our data suggests that RPN-12 is not essential for viability and lifespan of *C. elegans* under normal conditions (20ºC), but that absence of RPN-12 can result in a significant increase in mean lifespan under heat stress (25ºC).

## Methods

The *rpn-12(av93)* AG343 strain was generated via CRISPR/Cas9 genome editing technology following the direct delivery method developed by the Seydoux laboratory (Paix *et al.*, 2017). Two crRNAs (5’- agccagaagatttttatggg – 3’and 5’- actcgaacaaatcgtttaac – 3’) were used to guide the Cas9 to make cuts at specific regions of either ends of the *rpn-12* gene. An ssODN (5’-aaacattattggatttaagaaaatgtctgccgcccaaccggtgtctttcaagaactcaagcattgtattt-3’) was delivered as a template for the repair that results in a 952bp deletion that removes most of the first exon and then the entire second to last exons. Screening was performed using the co-conversion *dpy-10* method (Arribere *et al.*, 2014). Two independent strains of the *rpn-12* deletion were generated [*rpn-12(av93)* AG343 and *rpn-12(ana10)* WDC10] through the previously described method, and each strain outcrossed five times before experiments conducted. Both strains showed similar viability and fertility phenotypes, however only *rpn-12(av93)* was used for the lifespan experiments. Lifespan assays were conducted by placing age synchronized L4 larvae of *rpn-12(av93)* and wild type animals on MYOB plates seeded with OP50 (10-20 worms per plate). The worms were then passaged to new MYOB plates every two days. The number of dead hermaphrodites was scored every two days until all worms were dead and the percentage of surviving animals calculated. Missing worms were excluded from the statistical analysis.

## Reagents

AG343 *rpn-12(av93)*

WDC10 *rpn-12(ana10)*
